# 

**Published:** 1881-03

**Authors:** 


WHOLE NUMBER, CCXXXVI.

MARCH, 1881.

NUMBER 8, VOLUME XX.

INDEX MEDICUS.

### THE BUFFALO Medical and Surgical Journal.

S.G.O. INDEXED

EDITORS:

BUFFALO:

PUBLISHED BY THE MEDICAL JOURNAL ASSOCIATION.

**Read the Notice of this Journal among the Advertisements.**

Entered at the Post-Office at Buffalo, N. Y., as Second-Class Mail Matter.

